# Suckler Bulls Slaughtered at 15 Months of Age: Effect of Different Production Systems on the Fatty Acid Profile and Selected Quality Characteristics of *Longissimus Thoracis*

**DOI:** 10.3390/foods8070264

**Published:** 2019-07-18

**Authors:** Lara Moran, Shannon S. Wilson, Cormac K. McElhinney, Frank J. Monahan, Mark McGee, Maurice G. O’Sullivan, Edward G. O’Riordan, Joseph P. Kerry, Aidan P. Moloney

**Affiliations:** 1 Teagasc, Food Research Centre, Ashtown, Dublin 15, Ireland; 2 School of Food and Nutritional Sciences, University College Cork, Cork T12 XF62, Ireland; 3 Teagasc, Animal & Grassland Research and Innovation Centre, Grange, Dunsany, Co., Meath C15 PW93, Ireland; 4 School of Agriculture and Food Science, University College Dublin, Dublin 4, Ireland

**Keywords:** grass-fed, young bulls, nutritional quality, tenderness, carcass characteristics, pasture

## Abstract

The objective was to compare the quality of beef from bulls reared in typical Irish indoor systems or in novel grass-based systems. Bulls were assigned to one of the following systems: (a) grass silage plus barley-based concentrate ad libitum (CON); (b) grass silage ad libitum plus 5 kg of concentrate (SC); (c) grazed grass without supplementation (G0); (d) grazed grass plus 0.5 kg of the dietary dry matter intake as concentrate (GC) for (100 days) until slaughter (14.99 months). Carcass characteristics and pH decline were recorded. *Longissimus thoracis* was collected for analytical and sensory analysis. Lower carcass weight, conformation and fatness scores were found for grazing compared to CON and SC groups. CON bulls had highest intramuscular fat and lighter meat colour compared with grazing bulls. The SC meat (14 days aged) was rated higher for tenderness, texture, flavour and acceptability compared with grazing groups. CON saturated and monounsaturated fatty acid (FA) concentration was highest, conversely, omega-3 FA concentration was higher for GC compared with CON, while no differences were found in polyunsaturated FA. In conclusion, while market fatness specification was not reached by grazed grass treatments, beef eating quality was not detrimentally affected and nutritional quality was improved.

## 1. Introduction

Some European markets require bulls to be slaughtered at less than 16 months of age and to have a carcass fatness score of six (1–15 scale) [1]. These market specifications are presumed to reflect an effect of age and carcass fatness on meat quality and consumer preferences and are generally achieved by the use of high energy rations [1]. However, these specifications limit the ability of farmers to explore the use of alternative lower-cost feed options and to increase the profitability of the bull beef enterprise.

Feedstuff costs are a major proportion of the total expenditure in cattle production [2]. In regions with a temperate climate, grazed grass is a cheaper feedstuff [3,4]. Conserved grass (silage) has been used in this bull production system to achieve the carcass specification required but few meat quality studies are available. As grazed grass is cheaper than conserved grass, there is interest in including grazed grass in bull production, while the inclusion of a grazing period would also decrease the costs associated with cattle housing for a prolonged period.

Grass-fed beef is increasingly appreciated due to its “green-healthy image” also related to an idyllic image of animal production [5], which, in turn, can play a crucial role in consumer acceptability [6]. Diet conscious consumers will also prefer the overall lower fat content and altered fatty acid profile of a grass-fed beef product [7]. However, tenderness is the most important quality characteristic for the consumer [8,9] and beef from grass-fed bulls has been reported as being less tender than beef from grain-fed bulls [10]. While market specifications reflect real and perceived market preferences, ideally, they should be supported by scientific evidence. There is, however, a poor relationship between carcass quality as assessed by the European classification system (EUROP) and beef quality characteristics, particularly eating quality [11]. Consequently, modified lower-cost production systems need to be studied not only for achievement of current market specifications but also for meat quality characteristics and consumer preferences.

Therefore, the aim of the present study was to compare two typical Irish bull production systems with novel grass-based systems to determine whether the carcasses comply with the actual market requirements and to determine whether carcasses that did not achieve the fat score specification were indeed inferior with respect to quality and nutritional characteristics.

## 2. Materials and Methods

### 2.1. Animals, Management and Feed Analyses

The study was carried out under license from the Irish Government Department of Health and Children (held by Edward G. O’Riordan B100/2483) and all procedures used complied with national regulations concerning experimentation on farm animals.

Spring-born late-maturing breed (Limousin and Charolais) sired suckler male cattle (*n* = 60; 386, s.d. 28.0 kg live weight; 337, s.d. 36.3 days of age) were housed in a slatted floor shed and offered grass silage to appetite, supplemented with 2 kg of a barley/soya-based concentrate for eight weeks prior to the start of the experiment. At the end of the winter (11 February), the animals were blocked by breed, weight and age and randomly assigned to one of four dietary groups (*n* = 15): Two indoor diets: (a) barley-based concentrate comprised of 862 g/kg rolled barley, 60 g/kg soya bean, 50 g/kg cane molasses and 28 g/kg vitamins and minerals offered ad libitum plus grass silage offered ad libitum (CON) or (b) grass silage ad libitum supplemented with the above concentrate (SC) and two outdoors diets: (a) rotationally grazed grass without concentrate supplementation (G0) or (b) grazed grass plus 0.5 of the dietary dry matter (DM) intake as the above concentrate (GC).

Indoor animals had a lying area of c. 2.72 m^2^ per animal in a slatted, concrete floor shed. Beginning 23 February (considered as day 0 of the experiment), CON bulls were offered an increasing allowance of concentrates until the ad libitum level of consumption was achieved. The concentrate allowance for animals on SC was increased as the animals gained weight (4 kg, 5 kg, 6 kg and 6.5 kg per animal at days 0,10, 30, and 90 of the experiment, respectively). The grass silage (Table 1) was a first harvest from a predominantly perennial ryegrass sward, mowed and wilted for 24 h before ensiling. Grass silage and concentrates were offered separately.

Outdoors bulls remained on the pre-experimental ration until they were turned out to pasture (day 21 of the experiment) and rotationally grazed perennial ryegrass (*Lolium perenne* L.) dominant swards for 79 days. The total grazing area was a single block of 13.2 hectares (ha), split into three equal farmlets (4.4 ha). To ensure that the response to concentrates at pasture was not confounded with differences in herbage quality, the two grazing groups were allocated herbage of similar pre-grazing height and mass (2300 kg DM/ha) and the sub-paddock area was adjusted such that post-grazing sward height and herbage mass and, residency time were similar for each grazing treatment. The concentrate allowance, which averaged 5 kg per animal daily, was offered once daily and it was based on expected grass consumption as observed in previous studies with similar animals at this stage of the grass growing season. Based on previous research at this centre, initial grazing areas for GC were adjusted to be 0.73 of that of G0, and these proportions were altered during the experiment as necessary.

Samples of feeds were collected periodically throughout the study and analysed as previously described [12]. The chemical composition and fatty acid profile of the feedstuffs are shown in Table 1.

All animals were slaughtered on one day at 14.99 SD. 0.93 months of age with 53 of the 60 animals being under 16 months of age. The live weight was recorded on the farm on the morning of slaughter.

### 2.2. Slaughter, Sampling Procedures, PH and Colour Measurement

On the day of slaughter, the animals were transported approximately 30 km to a commercial slaughter plant and slaughtered immediately after arrival by bolt stunning followed by exsanguination from the jugular vein. Electrical stimulation was not applied, and the carcasses were hung by the Achilles tendon. The slaughter and dressing procedures were in accordance with European Union Regulations (EC) No. 1009/2009 and No. 853/2004 and carcass weight, conformation and fatness scores were recorded. Carcass grades for conformation and fatness were assessed using the numerical value within a 15-point scale [13]. Carcasses were then placed in a chiller set at 5 °C. Approximately 10 h later, the chiller temperature was set to 0 °C.

The pH and temperature of the *longissimus thoracis* (LT) at the 10th rib were recorded in the left side carcass at 1 h, 3 h, 5 h and 7 h post mortem, with a portable pH meter with temperature compensation (Model WP-80 (pH/ORP/T meter), TPS Pty, Ltd. Springwood, Queensland, Australia.) and a glass pH probe (Glass electrode: model EC-2010-06, Refex Sensors Ltd. Westport, Ireland.) using a scalpel incision for each measurement as described by Pearce et al. [14]. The pH meter was (re)calibrated at ambient temperature intermittently during the measurement period. The chill temperature was set to 0 °C approximately 12 h after the bulls were slaughtered.

After approximately 48 h in the chiller, carcasses were moved to the deboning hall (4 °C). The colour of the LT as Hunter lab values was measured at the 5th/6th rib interface, 1 h after cutting and exposure to air using a portable spectrophotometer (Miniscan EZ, HunterLab, Reston, VA, USA). The pH of LT was measured at this location as described above. The cube roll (commercial cut that begins between the 5th and 6th rib and ends between the 10th and 11th rib) was then removed, vacuum packed and transported to Teagasc, Food Research Centre, Ashtown, Dublin. Beginning at the 10th rib end, two steaks of 2.5 cm thick were stored at −20 °C for composition and fatty acid determination. The remainder of the cube roll was vacuum packed and wet-aged for 12 additional days (4 °C, in the dark) for a total of 14 days of ageing. Thereafter, it was sliced (2.5 cm thick steaks) for sensory evaluation, cook loss and instrumental texture analysis. All samples were then vacuum packed and frozen at −20 °C for subsequent analysis.

### 2.3. Meat Proximate Composition

Steaks for proximate analysis were thawed, trimmed of external fat and connective tissue, and the trimmed muscle was blended (R101, Robot Coupe SA, Vincennes Cedex, France). The moisture content of each sample was determined in duplicate using a microwave instrument (Microwave SMART Trac; CEM Corporation, Matthews, NC, USA) according to [15]. The intramuscular fat (IMF) content was measured as described by Folch et al. [16] (1957). Protein was determined in duplicate using a LECO protein analyser (Model FP-428, Leco Corporation, St. Joseph, MI, USA) based on the Dumas method [17].

### 2.4. Instrumental Texture and Sensory Evaluation

Frozen vacuum-packed steaks were thawed in circulating water at 20 °C. All external fat and connective tissue surrounding the muscle was removed, the steaks were conditioned for 15 min at 20 °C before cooking for sensory and instrumental texture analysis. After the excess moisture was removed, the weight of the steaks was recorded. The steaks were subsequently cooked in vacuum pack bags to an internal temperature of 70 °C, by immersing in a water bath (Model Y38, Grant Instruments Ltd. Royston, UK) at 72 °C. The internal temperature of the steaks was measured using a digital thermometer (HI 904, Hanna Foodcare Instruments, Bedfordshire, UK) [18]. After cooking, all the juices were poured out of the bag and the steaks were left to cool to room temperature, finally the weight of the cooked steak recorded. The cook loss (CL) was determined by the following formula:

CL% = (raw weight − cooked weight) ÷ raw weight × 100(1)

All steaks were stored in a closed bag and tempered overnight at 4 °C for subsequent Warner–Bratzler shear force (WBSF) analysis [18]. Six cores (1.25 cm diameter) parallel to the direction of the muscle fibres were obtained and sheared using an Instron Universal testing machine (model 5543, Instron Corporation. Bucks, UK) equipped with a Warner–Bratzler shearing device. The crosshead speed was 5 cm/min. Instron Series IX Automated Materials Testing System software for Windows (Instron Corporation. Bucks, UK) was employed in the analysis. Three parameters were used to define the instrumental texture of meat: WBSF (N) or peak strength or force required to shear through a meat sample; modulus of deformability (Mpa) or the slope between the 20% to 80% segment of the total peak; and total energy (J) or total peak area.

Sensory testing was conducted using untrained assessors (*n* = 15) [19,20] who ranged in age from 20–50 and who consumed beef regularly. Sensory analysis was carried out in the sensory kitchen in University College Cork. The kitchen features sensory booths and conforms to the standards of the International Organization for Standardization [21]. The analysis was conducted under standard lighting (LUX, 1000) in well-ventilated and portioned panel booths. Steaks were grilled to an internal temperature of 72 °C, assigned three-digit random codes and served to assessors as 1 cm² pieces, in randomised order [22]. Each assessor was asked to rate the sensory qualities of steak from each animal according to the methodology of the American Meat Science Association [18,23]. The assessors rated five sensory qualities on a scale (8-point hedonic) from 1–8 for tenderness (3–5 chews) where 1 = extremely tough and 8 = extremely tender, overall flavour where 1 = very poor and 8 = extremely good, overall firmness where 1 = extremely mushy and 8 = extremely firm, overall texture where 1 = very poor and 8 = extremely good and overall acceptability where 1 = not acceptable and 8 = extremely acceptable. Distilled water and unsalted soda crackers were provided to purge the palate of residual flavour notes between samples.

### 2.5. Meat Fatty Acid Analysis

Lipid extraction and fatty acid methylation was carried out as described by Noci et al. [12]. Fatty acid methlyesters (FAMEs) were analysed using a Varian 3500 GLC (Varian, Harbor City, CA, USA) fitted with a flame ionization detector. All samples were methylated in duplicate and each sample was injected, in splitless mode, twice onto the GLC column, using a Varian 8035 auto-sampler. Separation of the FAMEs was performed on a 100 m CP-Sil 88 column (100 m × 25 mm × 0.2 µm Supelco, Bellefonte, PA, USA) using H as the carrier gas. The GLC conditions have been described previously by Shingfield et al. [24]. Data were recorded and analysed on a Minichrom PC system (VG Data System, Manchester, UK). Individual FAMEs were identified by retention time with reference to the external standard (Supelco 37 component FAME Mix, Supelco Inc., Bellefonte, PA, USA) and quantified by using the internal standard C 23:0. The atherogenic index (AI) and thrombogenic index (TI) were calculated according to Ulbricht and Southgate [25].

### 2.6. Statistical Analysis

All data were subjected to analysis of variance (ANOVA) via the generalised linear mixed model, GLIMMIX procedure of SAS (SAS Inst. Inc., Cary, NC, USA) using block and production system as sources of variation. Animal was the experimental unit. The random tool of the GLIMMIX procedure was used to include “time” as a repeated measurement for the analysis of pH decline and assessor for the analysis of the sensory data. Data are presented as least squares means and when significant effects were detected, the post hoc Tukey test was used to separate the means. The level of significance used was *P* < 0.05.

The following models were used for instrumental and sensory measurements, respectively:

Y = µ + P + B + P * ID + ∑

Y = µ + P + B + C (ID) + ∑

µ = intercept; ∑ error. Fixed factors P: production system; B: animal block. Random effect: P * ID: Production System * Animal identity, C (ID): consumer within ID of the animal.

## 3. Results

Unless otherwise indicated, all stated differences are significant (*P* < 0.05).

### 3.1. Animal Performance, Meat Quality and Sensory Evaluation

Animal Performance, meat quality and sensory evaluation results are presented in Table 2.

Bulls fed on concentrates reached a higher live weight compared with SC which in turn was higher than GC and G0 which did not differ. Carcass weight and fat scores were higher for CON compared with SC which in turn were higher than GC and G0 which did not differ. Conformation scores were higher for CON compared to SC and GC which in turn were higher than G0.

The IMF concentration was higher for CON compared to SC which in turn was higher than GC and similar to G0 which did not differ. Protein concentration was lower for G0 compared with the other three diets. Moisture concentration was lowest for CON and highest for G0.

**Figure 1:**
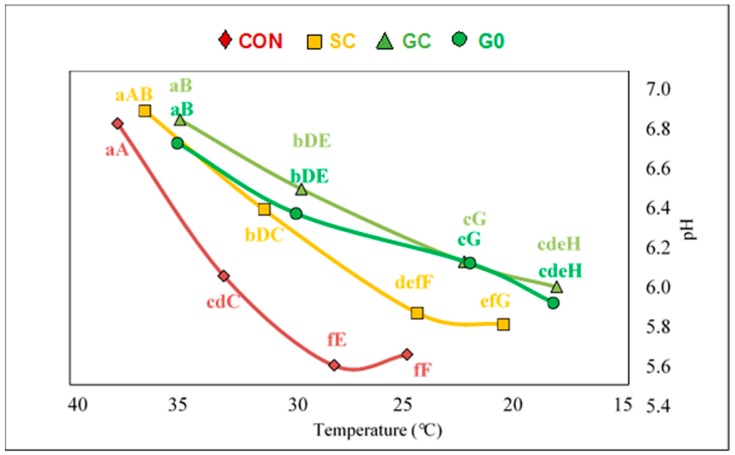
Pattern of pH/temperature decrease post-mortem (1, 3, 5 and 7 h) of longissimus muscle from bulls assigned to one of the following systems: grass silage plus barley-based concentrate ad libitum (CON); grass silage ad libitum plus 5 kg of concentrate (SC); grazed grass without supplementation (G0) or grazed grass plus 0.5 kg of the dietary dry matter intake as concentrate (GC) until slaughter at 15 months. ^a,b,c,d,e,f^ Different lowercase letters indicate statistical differences in pH (*P* < 0.05). ^A,B,C,D,E,F,G,H^ Different uppercase letters indicate statistical differences in temperature (*P* < 0.05).

Figure 1 shows the evolution of pH/temperature of LT up to 7 h post-mortem. No significant differences were found between treatments at 1 h post-mortem. Thereafter, the decrease in pH was more pronounced for CON than for the grazing groups. The SC pH decline was similar to that from grazing animals from 1–3 h post-mortem, while the pH for SC after 5 h was lower than in grazing animals and similar to CON. At 7 h post-mortem the pH for SC did not differ from the other groups. There was no difference between groups in ultimate pH (pHu) (Table 2). Muscle temperature decline (Figure 1) was less pronounced in CON animals than in grazing animals with SC intermediate. Thus after 1 and 3 h, temperature for CON was higher than for grazing animals but similar to SC. At 5 and 7 h post-mortem temperature for CON was higher than for the other groups, while temperature for SC was higher than GC and G0 which did not differ.

With regard to colour (Table 2), CON meat was lighter (higher L value) than meat from SC, which in turn was lighter than that from GC and G0. No differences were found in lightness between grazing treatments. On the other hand, indoor treatments meat had higher redness (a value) than that from grazing treatment. Neither CON and SC, nor GC and G0 differ between them in a value. Finally, CON and SC meat showed similar yellowness in meat colour (b value), while meat form these two indoor treatments was more yellow than for GC treatment. G0 meat did not differ in yellowness from GC and SC treatments.

No differences (*P* < 0.05) were found after 14 days of ageing for any of the variables related to instrumental texture (WBSF, modulus and energy). On contrary, the sensory panel rated LT from SC higher than GC and G0 (which did not differ) but similar to CON, for tenderness and overall flavour. For overall firmness, LT from CON, SC and GC were rated similarly while LT from G0 was rated lower than CON and SC but similar to GC. For overall texture, LT from CON and SC were rated similarly, while SC was rated higher than GC and G0 which did not differ. For overall acceptability, meat from CON, GC and G0 were rated similarly, but lower than SC.

### 3.2. Fatty Acid Profile

Data on fatty acid classes and relevant nutritional ratios are summarised in Table 3. The total fatty acid concentration and the concentration of total saturated fatty acids (SFA), total monounsaturated fatty acids (MUFA) and cis-MUFA were higher for CON than SC which in turn was higher than GC and G0 which did not differ. The concentration of trans-MUFA was higher for CON than for grazed animals while SC was intermediate

The concentration of total omega 3 polyunsaturated fatty acids (n-3) PUFA was similar for G0 and GC which was higher than SC which in turn was higher than CON. The concentration of total omega 6 (n-6) PUFA was similar for CON, SC and GC but higher than G0. The concentration of highly unsaturated fatty acids (HUFA) was higher for SC, GC and G0 (which did not differ) than CON. The n6 n3 PUFA ratio increased in the order G0 < GC < SC < CON. Grazing per se increased the 18:1-trans11: 18:1-trans10 ratio compared to SC and CON which did not differ. The PUFA:SFA ratio was similar for GC and G0 but higher than SC which in turn was higher than CON. The AI was similar for GC and G0 but lower than SC which in turn was lower than CON. The TI was similar for GC and G0 but lower than SC and CON which did not differ.

More detailed fatty acid profile analysis results are in Table 4, Table 5 and Table 6 provided in the discussion section.

The proportion of individual SFA and dimethyl acetals (DMA) is summarised in Table 4. Of the main SFA detected, the proportions of C14:0 and C16:0 were lower in GC and G0 than SC which in turn was lower than CON. The proportion of C18:0 was not affected by treatment. The proportions of C16:0 and C18:0 DMA were higher in GC and G0 than SC which in turn was higher than CON. The proportion of C18:1 DMA was higher in GC and G0 than SC and CON.

The proportion of individual MUFA is summarised in Table 5. Of the main cis MUFA detected, the proportion of C16:1 and cis 9 C18:1 were lower in GC and G0 than SC which in turn was lower than CON. The proportion of cis 11 C18:1 was lower for G0 than GC, SC was similar to GC and G0 but lower than CON. Of the main trans MUFA detected, the proportion of trans 10 C18:1 was higher for CON than the other three groups which did not differ. The proportion of trans 11 C18:1 was similar for GC and G0 and higher than SC and CON which did not differ.

The proportion of individual PUFA is summarised in Table 6. Of the main n-6 PUFA detected, the proportion of C18:2 was similar in CON, SC and G0, while GC was higher than SC and CON. The proportion of C20:4 was higher in GC and G0, which did not differ, than in SC which in turn was higher than CON. Of the main n-3 PUFA detected, the proportions of C18:3, C22:5 and C20: 5 were higher in GC and G0, which did not differ, than in SC which in turn was higher than CON.

## 4. Discussion

### 4.1. Carcass Characteristics

In this study, we considered the market specification that bulls must be younger than 16 months of age at slaughter to be the element of the production system that could not be changed. The context of the study therefore was to compare cheaper alternative systems with systems demonstrated to achieve the carcass weight and fat cover specifications [1]. The results indicate that the only grass-based system that reached the current market requirement was SC, i.e., the ration based on grass silage and concentrates, offered indoors with associated costs of housing. Therefore, the production of late-maturing sired bulls for slaughter at under 16 months of age from pasture seems not to be an option for meeting current market requirements. The age and fat score specification likely reflect a perception that meat from leaner and older animals is inferior in some quality characteristics. However, French et al. [26] reported a poor correlation between fatness scores and meat quality which was recently supported by Bonny et al. [11] from a much larger dataset. The primary objective of this study was to determine whether carcasses that did not achieve the fat score specification and were deemed too lean were indeed inferior with respect to quality for the consumer

### 4.2. Meat Quality

Mean carcass weights differed in this study and we acknowledge that carcass weight can influence meat characteristics [27]. In the current study, muscle from the grazing systems had lower IMF and lower protein in line with previous studies [10,28]. Banović et al. [29] reported that consumers pay more attention and choose more often meat products with lower fat content while Grunert [30,31] reported that visual fat has generally a negative effect on purchase decision. Therefore, while the leanness of beef cuts from grazing cattle may be appreciated by the consumer as a reduced fat product, low fat accretion in the animal will lead to a negative carcass classification based on EUROP scheme [32]. Thus, unlike the carcass grading system used in Australia [32], the lack of a relationship between carcass grading and eating quality in Europe is to the detriment of the primary producer and consumer.

The ultimate pH in the current investigation was within the normal range (5.4–5.8) described by Viljoen et al. [33] and agrees with previous studies [10,34]. Some authors have linked a higher pH in muscle from pasture animals with lower glycogen stores in muscle and/or increased stress during transport and slaughter management, since outdoor animals are not accustomed to human handling [27]. The latter is especially important in bulls, as male animals are more prone to suffer stress when forced into close social contact with their cohorts during transportation and lairage [35,36]. With correct management, normal ultimate pH can be achieved in under 16 months bulls independently of the dietary treatment.

The darker meat in the grazing groups is in line with previous studies [37,38]. Why SC was also darker than CON is not clear, since ultimate pH was similar, and the animals were managed similarly indoors. Darker colour may also be related to a higher myoglobin concentration in grass-fed animals. However, the redness (a) value indicates less red meat from grazed animals, which suggests that a higher pigment concentration is unlikely. However, myoglobin concentration measurement is needed to confirm this point.

In the present study, the steaks were aged for exactly 14 days as this is common industry practice. The lack of differences in the instrumental texture variables (WBSF, Modulus and Energy) agrees with those authors [39] who indicate that after 12 day of ageing differences in instrumental texture between factors such as gender or breed disappear [38,39].

Despite the lack of difference between treatments in instrumental texture, meat from SC was rated more highly by the sensory panel than meat from GC. Inconsistency between sensory and instrumental measurement results is common and is reflected in the modest correlation between both methods as reported by others [40,41]. Similar to the present study, Hedrick et al. [42] found that meat from cattle finished on silage was as tender or more tender than grain-finished cattle. The detrimental effect of concentrate supplementation outdoors compared to indoors (GC v SC) on tenderness was observed previously in 19-month-old bulls [34], albeit overall acceptability did not differ in that study.

The relationship of the rate and extent of post-mortem proteolysis with temperature and pH decline have been previously described [43]. Therefore, at the same chilling temperature, carcass fat cover impacts temperature and pH decline and sensory evaluation as previously highlighted [38,44,45].

The flavour of meat is dependent on the volatile profile [46] which in turn is greatly influenced by fat level and fatty acid composition [47,48]. Grass-produced beef can have a slightly less intense flavour than grain-produced beef [48], since higher levels of fat are associated with higher intensity of flavour. However, in the present experiment, flavour intensity was not assessed. In general, the effect of ration composition on flavour likeness results are inconsistent [38,49]. The higher rating in flavour for SC may be related to its higher fat content compared to outdoors animal and its lower SFA proportion compared to CON. This may give SC the best combination of IMF and fatty acid composition to meet consumer expectations. Overall, the lack of difference in sensory characteristics between CON and G0 is particularly noteworthy as G0 carcasses would be severely discounted relative to CON carcasses under the current EUROP grading system.

### 4.3. Fatty Acid Profile

Consumers are increasingly aware of the relationships between diet, health and well-being and this has resulted in a growing preference for foods which are healthier and more nutritious [50,51]. Some consumers prefer to purchase beef from grass fed cattle as meat and meat products from grass fed animals are often perceived as having higher amounts of nutritionally important compounds when compared with beef from non-grass-based production systems. In addition, ‘grass fed’ has been used to promote perception of animal health and well-being, and environmental sustainability [52]. Accordingly, the fatty acid composition was measured in beef from the contrasting production systems in this study. The higher concentration of fatty acids in the CON group per se reflects the higher energy consumption and associated fat deposition and is a consequence of this production system. The higher fatty acid concentration is the sum of higher concentrations of individual classes of fatty acids. When comparing studies in the literature with respect to the fatty acid profile of beef, it is important to be aware of differences in carcass weight or fatness between treatments. The higher concentration of SFA in CON animals compared to grass-fed animals in this study agrees with Aldai et al. [53] while other authors found no differences in SFA between grass and grain-fed meat [54].

An increase in IMF concentration on a common diet can alter the proportions of fatty acids, generally increasing MUFA and decreasing PUFA proportions [55,56]. Therefore, in this study the individual fatty acid data are presented as a proportion of total fatty acids to gain a better insight into any changes in the pattern or profile of fatty acids due to the different production systems examined.

The change in the individual SFA profile is generally in agreement with that reported by Alfaia et al. [54] and Aldai et al. [53]. The change in the SFA profile also reflects the fatty acid composition of the feedstuffs consumed, particularly the concentrate rations used. The lack of differences in total SFA concentration and in the SFA profile between both grazed grass-based diets (GC and G0) is in line with French et al. [28].

Three DMA derived from plasmalogen lipids were detected (16:0, 18:0 and 18:1). These DMA are generated from the vinyl chain linked at sn-1 position of plasmalogens, which are a particular class of glycerophospholipids present in cell membranes [57]. The lower proportions of DMA in muscle from indoor cattle (Table 4) agree with Aldai et al. [53]. Few studies have examined the nutritional importance of DMA in beef but DMA deficiency in humans has been associated with some diseases [57].

The higher concentration of MUFA in CON is consistent with previous investigations [53,54]. The alteration in the isomer profile of trans C18:1 observed in the present study has been previously reported [53,58]. A decrease in the trans-11 trans-10 18:1 ratio has been related with increased atherogenicity in animal models, while a higher ratio is related with increased rumenic acid (conjugated linoleic acid, see below) which has putative human health properties [59]. Changes in the proportions of these isomers has been suggested to reflect changes in rumen microbiota characteristics due to alteration in the rate of fermentation of the diet and associated changes in ruminal pH. C18:1-t11 rather than C18:1-t10 production in the rumen is associated with *Butyrivibrio fibrisolvens* which predominates in the rumen forage-finished animals [60]. While generally, a higher concentration of PUFA is observed in grass-fed beef compared to concentrate-fed beef [28,54,61], the lack of difference in the current study is likely related to the higher IMF concentration in CON. Also, many studies report fatty acid on a proportional basis. The proportions of PUFA (10.8, 15.3, 23.7 and 21.3 g/100 g fatty acids for CON, SC, GC and G0, respectively) in the present study support the literature but are high which likely reflects the very low total fatty acid concentration and very low neutral lipid proportion particularly in the grass-based production systems. In this regard, Nian et al. [62] reported a PUFA proportion of 23.5 g/100 g fatty acids for muscle from dairy origin bulls that had an IMF concentration of 0.5%, consistent with our data.

Linolenic acid (C18:3, omega 3) and linoleic acid (18:2n-6, omega 6) were the major PUFA of the omega series identified in this study. The omega series fatty acids cannot be synthesised by humans and are considered essential nutrients for humans [63]. The ratio is also considered important for human health since an excess of one family of omega 3 or omega 6 can interfere with the metabolism of the other [63]. It has been suggested that a healthy diet should have an n-6:n3 PUFA ratio higher than 4 [64], this ratio was only observed for outdoors animals suggesting that grass-fed beef has a higher nutritional value. For omega-3 fatty acids, EU (2010) states that a claim that a food is a source of omega-3 fatty acids may only be made where the product contains at least 300 mg α-linolenic acid per 100 g). The concentrations of linolenic acid in the present study were 9, 13, 17 and 18 mg/100 g muscle for CON, SC, GC and G0, respectively. None of the beef in this study meets this claim.

Higher linoleic acid in GC and G0 compared with CO animals is unusual based on the literature, but may reflect the extremely low IMF concentration in these groups. The higher proportion of linolenic acid its elongation products mainly C22-5n-3 and C20:5n-3 in GC and G0 is consistent with the literature [28].

Two CLA isomers were detected in the present study, the major isomer rumenic acid (C18:2-c9,t11) and the minor isomer, C18:2 t10c12. The higher rumenic acid proportion in GC and G0 is also consistent with the literature [28,53]. Animal studies demonstrated that CLA can reduce carcinogenesis, atherosclerosis and diabetes [65,66,67]. However, due to the higher IMF concentration in CON and SC compared to GC and G0 (Table 6), an individual consuming 100 g beef would consume a similar amount of rumenic acid from beef from all the production systems examined.

Since humans are able to transform trans vaccenic acid (C18:1-t11) into rumenic acid (9c11t-C18:2) at a rate between 5 to 12% [68], the higher concentration of trans vaccenic acid (6.3, 5.3, 7.0 and 7.5 mg/100 g muscle for CON, SC, GC and G0, respectively) would also contribute to a nutritional enhancement of beef from the grass-based production systems. We acknowledge that the proportion of CLA found in the present study was low compared with other studies, however the levels are similar to those reported by Aldai et al. [53], who explained the lower CLA concentration on the basis of the late-maturing breeds used.

The HUFA meat content is also an important nutritional factor, since the human efficiency of transforming α-linolenic acid to EPA (eicosapentaenoic acid C20:5n-3), DPA (docosapentaenoic acid C22:5n-3) and DHA (docosahexaenoic acid C22:6 n-3) is very low. On the other hand, HUFA (especially DHA and EPA) have been related with the prevention of atherosclerosis, heart attack, depression and cancer [69]. The higher concentration of HUFA and proportions of EPA and DPA in GC and G0 is similar to Alfaia et al. [54]. For longer carbon chain omega-3 fatty acids, EU (2010) states that a claim that a food is a source of omega-3 fatty acids may only be made where the product contains at least 40 mg of the sum of EPA and DHA per 100 g.). In the present study, EPA + DHA concentrations were 9.5, 14.4, 14.0 and 14.5 mg/100 g muscle for CON, SC, GC and G0, respectively. None of the beef in this study meets this claim.

In line with previous results, the nutritional indices, AI and TI, were better for grass-fed beef compared to CON indicating a general improvement of the nutritional quality. Similarly, the PUFA:SFA ratio is suggested to be above 0.4 [70] and only the grass-based production systems achieved this target.

## 5. Conclusions

In conclusion, while only the indoor production systems met the market carcass fat specifications, beef eating quality of grass-fed animals was not detrimentally affected. This, together with the fact that grass-beef systems result in leaner meat with a fatty acid profile better for the health of the consumer, makes these “grass-based” productions systems a feasible alternative especially for “health-concerned” consumers. It is clear that the carcass fat specifications required by the industry are not justified on an eating quality basis.

## Figures and Tables

**Table 1:** Chemical composition of the dietary ingredients.

	Grazed Grass	Grass Silage	Concentrates
Dry matter (DM) (g/kg fresh matter)	180	247	804
pH	−	−	3.92
DM digestibility (g/kg)	761	680	−
Composition of DM (g/kg)			
Starch	−	−	544
Ash	112	92	55
Crude protein	163	130	144
Neutral detergent fibre	468	577	185
Acid detergent fibre	−	−	67
Water soluble carbohydrate	123	−	−
Acid hydrolysis ether extract	−	−	31
Fatty acid profile (%)			
14:0	0.11	0.36	0.21
16:0	18.18	15.59	22.12
16:1	0.00	0.05	0.12
17:0	0.09	0.04	0.08
18:0	2.32	1.86	1.61
18:1-c11	0.18	0.69	0.76
18:1-nc9	3.00	2.77	14.33
18:2-nc6	13.49	17.50	54.06
18:3-n3	55.67	56.52	4.44
20:0	0.19	0.29	0.17
20:1	0.00	0.00	0.68
20:5-n3	0.51	0.00	0.32
21:0	0.06	0.00	0.00
22:0	0.66	0.96	0.24
22:1-n9	0.07	0.06	0.08
24:0	0.89	0.84	0.13
Others	4.52	2.47	0.58

**Table 2:** Animal performance and characteristics of *longissimus thoracis* muscle from bulls assigned to one of the following systems: grass silage plus barley-based concentrate ad libitum (CON); grass silage ad libitum plus 5 kg of concentrate (SC); grazed grass without supplementation (G0) or grazed grass plus 0.5 kg of the dietary dry matter intake as concentrate (GC) until slaughter at 15 months.

Variable	CON	SC	GC	G0	SEM	*P*-Value
BW (kg)	604 ^a^	557 ^b^	515 ^c^	498 ^c^	7.135	<0.001
Average daily weight (kg/day)	2.00 ^a^	1.58 ^b^	1.47 ^b,c^	1.30 ^c^	0.048	<0.001
Carcass weight (kg)	358 ^a^	314 ^b^	288 ^c^	277 ^c^	4.997	<0.001
Kill out proportion (%)	59.2 ^a^	56.5 ^b^	56.0 ^b^	55.7 ^b^	0.320	<0.001
Fat score (1–15)	7.27 ^a^	6.00 ^b^	4.27 ^c^	3.67 ^c^	0.231	<0.001
Conformation (1–15)	9.93 ^a^	8.53 ^b^	8.40 ^b^	7.67 ^c^	0.168	<0.001
Crude protein (%)	23.0 ^a^	22.9 ^a^	22.7 ^a^	21.6 ^b^	0.191	0.039
Moisture (%)	74.8 ^c^	75.2 ^b,c^	76.1 ^a,b^	76.7 ^a^	0.178	<0.001
Intramuscular fat (g/100 g meat)	2.20 ^a^	1.85 ^b^	1.48 ^c^	1.59 ^b,c^	0.063	<0.001
Ultimate pH	5.60	5.60	5.62	5.62	0.006	0.323
L	37.2 ^a^	34.7 ^b^	31.7 ^c^	32.5 ^c^	0.390	<0.001
a	19.3 ^a^	19.2 ^a^	18.3 ^b^	18.4 ^b^	0.139	0.022
b	12.7 ^a,b^	12.8 ^a^	11.8 ^c^	12.2 ^b,c^	0.096	0.001
Cook loss (%)	25.7	27.7	28.6	27.6	0.369	0.056
Warner–Bratzler shear force (N)	35.8	33.6	35.3	32.5	0.936	0.587
Slope (MPa)	0.785	0.770	0.845	0.733	0.024	0.452
Energy (J)	0.230	0.229	0.237	0.219	0.006	0.733
Tenderness (1–8)	4.70 ^a,b^	5.22 ^a^	4.24 ^b^	4.60 ^b^	0.109	<0.001
Overall flavour (1–8)	5.39 ^a,b^	5.74 ^a^	5.02 ^b^	5.22 ^b^	0.076	0.001
Overall firmness (1–8)	5.39 ^a^	5.11 ^a^	5.02 ^a,b^	4.69 ^b^	0.080	0.001
Overall texture (1–8)	5.02 ^a,b^	5.42 ^a^	4.71 ^b^	4.81 ^b^	0.087	0.001
Overall acceptability (1–8)	5.10 ^b^	5.56 ^a^	4.79 ^b^	5.02 ^b^	0.084	0.001

^a,b,c^ Values within a row with different superscript differ significantly at *P* < 0.05.

**Table 3:** Summary of total fatty acids (total FA mg/100 g muscle), and nutritional ratios of the *longissimus thoracis* muscle from bulls assigned to one of the following systems: grass silage plus barley-based concentrate ad libitum (CON); grass silage ad libitum plus 5 kg of concentrate (SC); grazed grass without supplementation (G0) or grazed grass plus 0.5 kg of the dietary dry matter intake as concentrate (GC) until slaughter at 15 months.

	CON	SC	GC	G0	SEM	*P*-Value
∑FA	1155 ^a^	823 ^b^	558 ^c^	575 ^c^	47.9	<0.001
SFA	517 ^a^	340 ^b^	196 ^c^	208 ^c^	24.9	<0.001
MUFA	478 ^a^	308 ^b^	169 ^c^	180 ^c^	25.3	<0.001
PUFA	125	126	132	123	2.15	0.409
trans-MUFA	25.6 ^a^	17.4 ^b^	14.1 ^b^	16.2 ^b^	1.24	0.007
cis-MUFA	451 ^a^	291 ^b^	154 ^c^	164 ^c^	23.01	<0.001
PUFA-n3	26.4 ^c^	37.2 ^b^	43.9 ^a^	44.2 ^a^	1.31	<0.001
PUFA-n6	85.7 ^a^	77.4 ^a^	78.8 ^a^	67.7 ^b^	1.68	0.003
HUFA	35.5 ^b^	44.4 ^a^	47.0 ^a^	47.2 ^a^	1.10	0.001
CLA	3.62	2.46	2.15	2.94	0.212	0.076
n6/n3 PUFA	3.24 ^a^	2.16 ^b^	1.83 ^c^	1.55 ^d^	0.095	<0.001
t11/t10 18:1	0.860 ^b^	2.82 ^b^	11.67 ^a^	14.17 ^a^	1.131	<0.001
PUFA:SFA	0.268 ^c^	0.423 ^b^	0.702 ^a^	0.636 ^a^	0.031	<0.001
Atherogenic index	0.643 ^a^	0.553 ^b^	0.377 ^c^	0.408 ^c^	0.019	<0.001
Thrombogenic index	1.65 ^a^	1.63 ^a^	1.36 ^b^	1.45 ^b^	0.027	<0.001

SFA = saturated fatty acid; MUFA = monounsaturated fatty acids; PUFA = polyunsaturated fatty acids; HUFA = Highly unsaturated fatty acids larger than 19 carbons; CLA = conjugated linoleic acid; n6 = omega 6 fatty acid; n3 = omega 3 fatty acids ^a,b,c^ Values within a row with different superscript differ significantly at *P* < 0.05.

**Table 4:** Individual saturated fatty acid (SFA) and dimethyl acetal (DMA) proportions (of total fatty acids ^1^) in the *longissimus thoracis* muscle from bulls assigned to one of the following systems: grass silage plus barley-based concentrate ad libitum (CON); grass silage ad libitum plus 5 kg of concentrate (SC); grazed grass without supplementation (G0) or grazed grass plus 0.5 kg of the dietary dry matter intake as concentrate (GC) until slaughter at 15 months.

	CON	SC	GC	G0	SED	*P*-Value
SFA						
8:0	0.017 ^c^	0.064 ^b^	0.086 ^a^	0.096 ^a^	0.006	0.003
10:0	1.01 ^c^	1.31 ^b^	1.85 ^a^	1.93 ^a^	0.083	<0.001
11:0	0.125 ^b^	0.138 ^b^	0.242 ^a^	0.230 ^a^	0.013	<0.001
12:0	0.066 ^b^	0.097 ^b^	0.115 ^a,b^	0.141 ^a^	0.007	0.002
14:0	2.19 ^a^	1.65 ^b^	0.940 ^c^	1.04 ^c^	0.091	<0.001
iso-15:0	0.159 ^b^	0.222 ^a,b^	0.251 ^a^	0.277 ^a^	0.014	0.045
anteiso-15:0	0.162 ^b^	0.175 ^b^	0.218 ^a^	0.246 ^a^	0.007	<0.001
15:0	0.483	0.536	0.508	0.537	0.014	0.529
16:0	22.9 ^a^	20.1 ^b^	15.2 ^c^	15.5 ^c^	0.525	<0.001
iso-17:0 ^2^	1.12 ^a^	0.349 ^c^	0.875 ^b^	0.767 ^b^	0.054	<0.001
17:0	1.09 ^c^	0.905 ^b^	0.670 ^a^	0.697 ^a^	0.030	<0.001
18:0	13.9	14.2	13.1	13.4	0.191	0.229
19:0 ^3^	0.151	0.150	0.143	0.145	0.005	0.955
20:0	0.234 ^a^	0.113 ^c^	0.182 ^b^	0.197 ^b^	0.029	<0.001
24:0	0.107	0.131	0.100	0.098	0.030	0.975
DMA						
DMA 16:0	2.06 ^c^	3.14 ^b^	4.45 ^a^	4.75 ^a^	0.184	<0.001
DMA 18:0	1.20 ^c^	2.04 ^b^	2.90 ^a^	2.91 ^a^	0.122	<0.001
DMA 18:1	2.24 ^b^	2.97 ^b^	4.18 ^a^	4.55 ^a^	0.194	<0.001

^1^ Only values superior to 0.05 have been reported ^2^ coeluted with t9-16:1. ^3^ coeluted with c15-18:1. ^a,b,c^ values within a row with different superscript differ significantly at *P* < 005.

**Table 5:** Individual monounsaturated fatty acid (MUFA) proportion (of total fatty acids ^1^) in the *longissimus thoracis* muscle from bulls assigned to one of the following systems: grass silage plus barley-based concentrate ad libitum (CON); grass silage ad libitum plus 5 kg of concentrate (SC); grazed grass without supplementation (G0) or grazed grass plus 0.5 kg of the dietary dry matter intake as concentrate (GC) until slaughter at 15 months.

	CON	SC	GC	G0	SEM	*P*-Value
MUFA						
14:1	0.345 ^a^	0.230 ^b^	0.088 ^c^	0.103 ^c^	0.019	<0.001
cis MUFA						
c7-16:1	0.179 ^b^	1.30 ^a^	1.54 ^a^	1.80 ^a^	0.115	<0.001
c9-16:1	2.77 ^a^	2.21 ^b^	1.39 ^c^	1.47 ^c^	0.100	<0.001
c5-17:1	0.094 ^d^	0.226 ^v^	0.363 ^b^	0.504 ^a^	0.025	<0.001
c7-17.1	0.110 ^c^	0.633 ^b^	0.518 ^a^	0.518 ^a^	0.029	<0.001
c9-17:1	0.700 ^a^	0.383 ^c^	0.602 ^a,b^	0.534 ^b^	0.024	<0.001
c10-17:1	0.303 ^a^	0.001 ^b^	0.093 ^b^	<0.001 ^b^	0.022	<0.001
c11-17:1	0.798 ^b^	1.03 ^b^	1.52 ^a^	1.74 ^a^	0.075	<0.001
c9-18:1	27.9 ^a^	23.0 ^b^	16.2 ^c^	16.1 ^c^	0.854	<0.001
c11-18:1	1.53 ^a^	1.28 ^b,c^	1.36 ^b^	1.19 ^c^	0.029	0.001
c12-18:1	0.314	0.389	0.376	0.275	0.023	0.276
c13-18:1	0.187 ^a^	0.130 ^b^	0.098 ^b,c^	0.085 ^c^	0.010	<0.001
c14-18:1	0.046	0.131	0.090	0.100	0.012	0.144
c16-18:1	0.106 ^a^	0.105 ^a^	0.072 ^b^	0.077 ^b^	0.005	0.010
trans MUFA						
t6+t8-18:1	0.145	0.112	0.105	0.090	0.006	0.064
t9-18:1	0.145	0.144	0.115	0.143	0.006	0.173
t10-18:1	0.726 ^a^	0.397 ^b^	0.230 ^b^	0.253 ^b^	0.062	0.023
t11-18:1	0.546 ^b^	0.644 ^b^	1.26 ^a^	1.31 ^a^	0.084	<0.001
t12+ t13-18:1	0.159 ^a^	0.132 ^a,b^	0.092 ^b^	0.103 ^b^	0.007	0.028
t15-18:1 ^2^	0.024 ^b^	0.084 ^a^	0.197 ^a^	0.343 ^a^	0.039	0.017
t16-18:1	0.205 ^a^	0.102 ^b^	0.097 ^b^	0.085 ^b^	0.013	0.070
n-9						
20:1n-9	0.147	0.136	0.122	0.123	0.005	0.291
22:1n-9	0.053 ^b^	0.114 ^a^	0.131 ^a^	0.107 ^a^	0.008	0.013
24:1n-9	0.069	0.061	0.122	0.092	0.020	0.639

n-9: Monounsaturated fatty acid omega 9; t: trans isomer, c: cis isomer ^1^ Only values superior to 0.05 have been reported ^2^ coeluted with c10-18:1. ^a,b,c^ Values within a row with different superscript differ significantly at *P* < 0.05.

**Table 6:** Individual polyunsaturated fatty acid (PUFA) proportions (of total fatty acids ^1^) in the *longissimus thoracis* muscle from bulls assigned to one of the following systems: grass silage plus barley-based concentrate ad libitum (CON); grass silage ad libitum plus 5 kg of concentrate (SC); grazed grass without supplementation (G0) or grazed grass plus 0.5 kg of the dietary dry matter intake as concentrate (GC) until slaughter at 15 months.

	CON	SC	GC	G0	SEM	*P*-Value
PUFA						
20:2	0.071 ^c^	0.109 ^a,b^	0.130 ^a^	0.092 ^b^	0.006	0.009
22:2	0.132 ^b^	0.182 ^b^	0.271 ^a^	0.267 ^a^	0.014	<0.001
Non conjugated 18:2						
9t,12t-18:2	0.128	0.110	0.107	0.094	0.006	0.444
t10,c15-18:2	0.133	0.131	0.123	0.148	0.006	0.612
t11,c15-18:2	0.127	0.159	0.149	0.132	0.009	0.555
c9,c15-18:2 ^2^	0.214 ^b^	0.254 ^b^	0.341 ^a^	0.348 ^a^	0.013	<0.001
CLA						
c9,t11-CLA	0.286 ^b^	0.279 ^b^	0.364 ^a,b^	0.471 ^a^	0.021	0.003
t10,c12-CLA	0.025	0.008	0.010	0.009	0.003	0.167
n-6						
18:2n-c6	6.04 ^b^	6.95 ^b^	9.94 ^a^	8.04 ^a,b^	0.338	<0.001
18:3n-6	0.063 ^b^	0.100 ^b^	0.216 ^a^	0.190 ^a^	0.016	0.004
20:3n-6	0.421 ^c^	0.558 ^b^	0.804 ^a^	0.714 ^a^	0.029	<0.001
20:4n-6	1.58 ^c^	2.32 ^b^	3.30 ^a^	3.30 ^a^	0.131	<0.001
n-3						
18:3n-3	0.790 ^c^	1.58 ^b^	3.06 ^a^	3.15 ^a^	0.150	<0.001
20:3n-3	0.076	0.046	0.087	0.069	0.007	0.130
22:5n-3	0.718 ^c^	1.30 ^b^	2.06 ^a^	2.15 ^a^	0.127	<0.001
22:6n-3	0.106	0.461	0.444	0.365	0.068	0.315
20:5n-3	0.718 ^c^	1.29 ^b,a^	2.06 ^a^	2.15 ^a^	0.102	<0.001
n-9						
20:3n-9	0.135 ^d^	0.229 ^c^	0.340 ^b^	0.398 ^a^	0.016	<0.001

CLA = conjugated linoleic acid. n-6 = polyunsaturated fatty acids omega 6; n-3 = polyunsaturated fatty acids omega 3; n-9 = polyunsaturated fatty acids omega 9; t = trans isomer, c = cis isomer ^1^ Only values superior to 0.05 have been reported; ^2^ coeluted with c9-19: ^a,b,c^ Values within a row with different superscript differ significantly at *P* < 0.05.
